# Open Hardware for Microfluidics: Exploiting Raspberry Pi Singleboard Computer and Camera Systems for Customisable Laboratory Instrumentation

**DOI:** 10.3390/bios13100948

**Published:** 2023-10-23

**Authors:** Rüya Meltem Sarıyer, Alexander Daniel Edwards, Sarah Helen Needs

**Affiliations:** 1 School of Pharmacy, University of Reading, Reading RG6 6DX, UK; r.sariyer@pgr.reading.ac.uk; 2 School of Electronics and Computer Science, University of Southampton, Southampton SO17 1BJ, UK

**Keywords:** Raspberry Pi, microfluidics, image detection, open source

## Abstract

The integration of Raspberry Pi miniature computer systems with microfluidics has revolutionised the development of low-cost and customizable analytical systems in life science laboratories. This review explores the applications of Raspberry Pi in microfluidics, with a focus on imaging, including microscopy and automated image capture. By leveraging the low cost, flexibility and accessibility of Raspberry Pi components, high-resolution imaging and analysis have been achieved in direct mammalian and bacterial cellular imaging and a plethora of image-based biochemical and molecular assays, from immunoassays, through microbial growth, to nucleic acid methods such as real-time-qPCR. The control of image capture permitted by Raspberry Pi hardware can also be combined with onboard image analysis. Open-source hardware offers an opportunity to develop complex laboratory instrumentation systems at a fraction of the cost of commercial equipment and, importantly, offers an opportunity for complete customisation to meet the users’ needs. However, these benefits come with a trade-off: challenges remain for those wishing to incorporate open-source hardware equipment in their own work, including requirements for construction and operator skill, the need for good documentation and the availability of rapid prototyping such as 3D printing plus other components. These advances in open-source hardware have the potential to improve the efficiency, accessibility, and cost-effectiveness of microfluidic-based experiments and applications.

## 1. Introduction

Open-source hardware has gained increasing attention in multiple scientific fields and notably in microfluidic lab-on-a-chip research and applications [1]. Free open-source software and hardware (FOSS and FOSH) now offer the opportunity to develop affordable scientific devices and laboratory equipment [2,3,4] in-house. FOSS is software in source code format that people can use, review, modify, and improve with or without certain license restrictions [5]. FOSH, on the other hand, provides information about the bill of materials, technical and user instructions, 3D CAD designs and all the information needed to rebuild a device [5,6]. The growth of FOSH and its use in research laboratories has increased in recent years, and it is currently following the rise of FOSS with a 20-year lag [6]. This provides an opportunity to reduce experimental costs for science and engineering [3], but the transparency and open publication of instrument design offers also benefits in research reproducibility. Such open-source materials could make scientific equipment much more accessible for research and the researcher [5]. In short, this open-source approach allows scientists to share, use and improve their hardware and software designs, with the aim of helping rapid scientific progress [7]. Alongside the use of open-source hardware and rapid prototyping tools such as 3D printing [8,9,10,11], the rise in the use of low-cost single-board computers has seen the Raspberry Pi become popular amongst a variety of disciplines within life sciences. A notable example, which is the focus of this review, is the use of Raspberry Pi hardware for microfluidics. Microfluidic device developers require hardware and software to build custom detection systems and for the analysis and interpretation of signals.

### Potential Benefits and Limitations of Open-Source Technologies

FOSH and FOSS technologies have some advantages compared to traditional “closed” or proprietary product designs: (a) in addition to being free of charge, the designs are developed by users who have strived to ensure the instrument is appropriate to a particular scientific task or application; (b) building your own experimental equipment provides a more comprehensive understanding of the principles behind the design, and better recognise any analytical limitations of such equipment; (c) manufacturing is immediate and local, empowering laboratories worldwide and potentially avoiding dependence on commercial instrument suppliers; and (d) open-source hardware and manufacturing has become a global phenomenon that allows the hiring of gifted hardware designers and software programmers to assist in creating cutting-edge products [6,12]. The community around open-source hardware can be found building detailed written and video tutorials online, allowing non-experts to recreate and remix designs.

**Figure 1:**
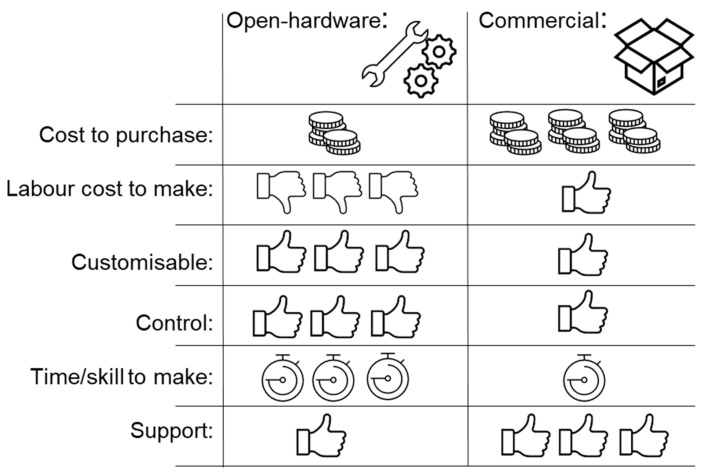
Benefits and limitations of open hardware.

However, a FOSH/FOSS equivalent is not available for all proprietary scientific equipment. Furthermore, not every scientific group is capable of manufacturing, using or maintaining open-source devices or software; this limitation may be further limited by the availability of good documentation (Figure 1). Finally, quality assurance and reliability are major difficulties for manufacturing FOSH [6].

## 2. Raspberry Pi/Pi Camera System

The 21st century commenced with the ascent of open-source and cost-effective software/hardware solutions, leading to the introduction of electronic prototyping tools like the Arduino microcontroller board for applications in fluid flow control systems [13,14,15,16,17,18,19,20] and droplet microfluidics [21], along with the Raspberry Pi single-board computers [22] used in colour and fluorescence detection, direct cellular image-detection systems and microscopy, and droplet microfluidics. Open-source hardware has been used for several aspects of microfluidic research and development, including pump and flow systems [13,14,15,16,17,19,20,23,24,25,26,27,28,29] and temperature control [30]. However, imaging is a key component of biological research—from microscopy to tracking optical readouts—and although there are many diverse approaches to sensing of biosystems, optical-based biosensors are one of the most widely used [30,31]. While many microfluidic systems intended for portable/low-cost applications are optimised to be read by eye, many require more complex optics, include features too small for direct visual readout, or benefit from digitisation and quantitation. Raspberry Pi, a low-cost mini-computer with accessories, has been increasingly frequently used in biological research [32], attracting attention especially with its different camera modules (GBP 25 to GBP 60) [1].

**Figure 2:**
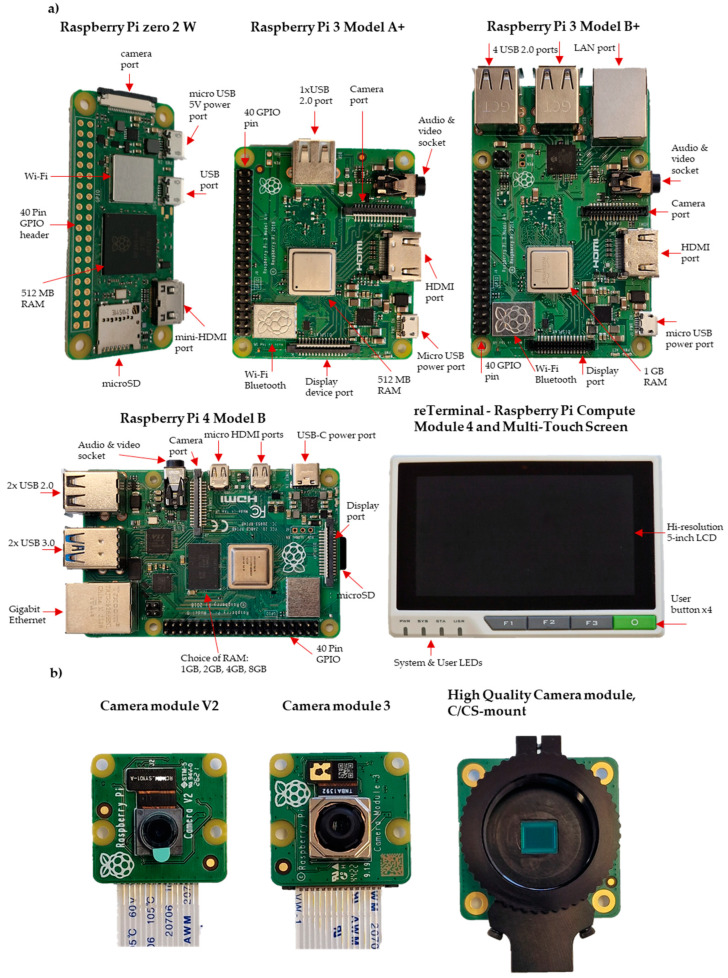
Photographs of (**a**) Raspberry Pi Boards and (**b**) Raspberry Pi Camera modules.

Raspberry Pi mini-computers offer different features that can be exploited for a variety of applications (Table 1, Figure 2). We can list these mini-computers as Raspberry Pi Pico (GBP 4.80), which is a small, fast and versatile board built using the RP2040 microcontroller chip; Pi Zero (GBP 5); Pi Zero W (GBP 15), which expands the Zero family with wireless LAN and Bluetooth connectivity; Pi Zero 2 W (GBP 20.50); Pi 1 Model A+ (GBP 20); Pi 3 Model A+ (GBP 25); Pi 3 Model B (GBP 40), which comes with 1.4 GHz 64-bit quad-core processor, dual-band wireless LAN, and Bluetooth 4.2/BLE features; and finally the Pi 4 (GBP 35 to GBP 75), which comes with a 1.5 GHz quad-core CPU and offers four different options, 1 GB, 2 GB, 4 GB, and 8 GB. The Pi 5, which will be released in October 2023, represents a substantial improvement over the Pi 4, boasting a faster processor, enhanced graphics, and speedier memory. The Pi 5 offers enhanced RAM with higher bandwidth and faster VideoCore VII GPU clocked at 800 MHz, supporting OpenGL ES 3.1 and Vulkan 1.2. It also incorporates a new image signal processor to handle camera data. Additionally, the Pi 5 increases its I/O bandwidth compared to the Pi 4. The microSD port on the Pi 5 supports HDR 104 high-speed mode with UHS-1 microSD cards. While the Pi 4 can read at 40–50 Mbps, the Pi 5 doubles the speed, reaching 80–90 Mbps. Moreover, the Pi 5 has two USB 3.0 ports, each with a dedicated 5 Gbps bandwidth. Additionally, the updated power supply provides extra amperage, increasing from 3A to 5A, allowing the Pi 5 to support more such devices [33].

One of the powerful features of these mini-computers is the general-purpose input/output (GPIO) pins lined up along the top edge of the board (Figure 2). There are two 5 V pins and two 3.3 V pins, as well as a set of ground pins (0 V) on the board. All remaining pins are general-purpose 3.3 V pins, and any of these pins can be assigned to the input or output pin on the software and can be used for various purposes [34]. Imaging usually requires lighting, but the built-in power pins on the Raspberry Pi are limited to only 5 V. In most cases, an external power supply is required for lighting.

Various camera modules are available from Raspberry Pi (Table 2, Figure 2). The Raspberry Pi Camera Module 2 (GBP 28.50) replaced the original Camera Module, offering improved features and performance. The Pi Camera Module 2 NoIR (GBP 28.50) is designed for low-light conditions, utilising infrared technology for image capture. With a 12 MP sensor and autofocus capabilities, the Pi Camera Module 3 (GBP 36) provides versatile functionality. The Pi Global Shutter Camera (GBP 50.20) is specialised for capturing rapid motion with its 1.6-megapixel sensor. Lastly, the Pi High-Quality Camera (GBP 57) features a 12.3-megapixel Sony IMX477 sensor, back-illuminated sensor architecture, adjustable back focus, and compatibility with C/CS mount or M12 mount options, ensuring adaptability and versatility for different setups (Figure 2) [33].

The Raspberry Pi computer/camera system has been used in conjunction with microfluidics for different biological studies, ranging from colour and fluorescence detection [37,38,39,40]; for direct cellular image detection and microscopy [39,41,42,43,44,45,46]; for time-lapse imaging [47]; and for droplet control microfluidics [1,48,49,50,51] (Table 3).

## 3. Open-Source Technologies in Microfluidic Research

The development of microfluidic systems often requires the use of novel or customisable equipment such as imaging systems or other data capture, liquid handling, controllers, the actuation of valves and pumping, and analysis software. By leveraging open-source technologies, researchers can access and adapt these tools to meet their specific needs, enabling greater flexibility and potential for rapid advancements in both microfluidics research and utilisation of microfluidics for other life science research.

In biological science, image detection systems and microscopes are powerful tools that provide the visualisation of cells, structural analysis, and the interpretation of experimental outputs such as changes in colour, enhanced fluorescence, or luminescence, playing a vital role in the medical diagnosis and treatment of various diseases [45].

Microfluidic devices allow the processing of very small volumes of sample liquid, reducing biological waste and often reducing dead volume, and minimising the use of expensive reagents [1]. There are multiple potential benefits of the distinctive fluid mechanics at sub-millimetre-length scale that have been exploited to reduce assay time, for separation or compartmentalisation, and for many other methods. These miniature devices also address challenges including portability, cost, analysis speed, and the reliance on specialised personnel, offering a potential future of cutting-edge approaches for diagnostics and treatment [58]. Microfluidic devices have the potential to miniaturise complex fluid manipulation in low-cost small packages [26]. These devices have the potential to offer a decentralised diagnostic alternative with simplified operation and the ability to quantify physiological parameters, overcoming limitations associated with conventional manual laboratory-based systems [58]. Open-hardware technology has shown that, for many applications, the in-house development of bespoke instrumentation using readily available low-cost components and materials can increase the quality and numbe of data from developmental systems and prototype devices, thus speeding up development and delivering a stronger evidence base.

Of particular importance, FOSH has been used extensively in microfluidic research to demonstrate the potential for assay miniaturisation to deliver Point-of-Care (PoC) diagnostic tests. These include the detection of nucleic acid, proteins, live/dead bacterial pathogens, viruses and more. In this review, we consider the use of open-source imaging systems on microfluidic studies. With the design of certain accessory attachments to low-cost, high-quality cameras (typically based on inexpensive modules developed for consumer photography), these can be used to carry out and record the results of analytical biosensing, helped greatly by the ever-increasing capabilities, ease of use and availability, and computing power of single-board computers such as those from the Raspberry Pi family.

### 3.1. Colour and Fluorescence Detection in Microfluidics

In many systems, the detection of biomolecules occurs via the indirect detection of colour or fluorescence changes, with the most frequent examples including enzyme-linked immunosorbent assay (ELISA) and polymerase chain reaction (PCR) but also including many other biochemical reactions or interactions. These have included the detection of specific bacteria and pathogens [53], the presence of proteins or nucleic acid biomarkers [38,40,52], and the monitoring of mammalian [37] and bacterial cell growth [59]. These assays and biosensors can be miniaturised using microfluidics and then monitored using open-source hardware systems. Depending on the size and resolution required of the microfluidic system, different Raspberry Pi camera modules may be required. Including time-resolved imaging (video and time-lapse) allows improved sensing that can extract further information such as kinetics, delivering more information than single timed endpoints. In many examples, devices aimed at Point-of Care/field applications, colour changes, and therefore test results, may especially benefit from being read by eye, thereby avoiding the need for instrumentation. However, the use of imaging allows digitisation, result recording and potentially the quantitation of results.

Such colour-based microfluidic tests include a paper microfluidic system to detect the albumin-to-creatine ratio in urine. Regular urine examinations are crucial for identifying potential kidney and urinary system disorders [52]. While there are several different methods available to determine the concentration of creatinine (CRE) in human urine, such as gas chromatography, liquid chromatography–mass spectrometry, colour spectrophotometry and electrochemical sensors, there are also methods like high-performance liquid chromatography, capillary electrophoresis, chemiluminescence and light scattering to determine the concentration of albumin (ALB) in urine. However, due to it complexity, time-consuming nature, cost, and the need for large devices and the intervention of skilled technicians associated with both ALB and CRE concentrations detection using these analytical techniques, there is a significant need for more affordable, simpler, and portable solutions. The average price range for commercially available devices typically varies between USD 2000 and USD 200,000. For this reason, researchers have presented a platform utilising a paper-based microfluidic chip that integrates with a Raspberry Pi-based detection system. The new detection system, on the other hand, can be built for an average of USD 130. The chip contains two colour bars (2.5 × 2.5 mm) for the determination of ALB and CRE concentrations using the colour change upon the addition of the analyte to bromocresol blue and picric acid for the detection of ALB and CRE, respectively. These bars can be used for the assessment of ALB and CRE concentrations through a simple inspection by the naked eye; however, for accurate measurement, the RGB intensity signals of the reaction complexes were imaged using an eight-megapixel Raspberry Pi camera and RGB colour intensity of the images analysed using a Raspberry Pi computer-based system [52]. The system was fully contained in a handheld device consisting of a Raspberry Pi 4B model computer, a Raspberry Pi Camera, a temperature controller, a touch switch module, a voltage controller, two rectangular LED white light sources, and a lithium battery, with dimensions of approximately 150 mm × 75 mm × 40 mm and a weight of around 492 g. Additionally, the system featured an insertion slot for the sliding microchip, which had five layers and measured approximately 50 mm × 20 mm × 4.5 mm in size and a Raspberry Pi smart tablet positioned on the detection box to visualise the detection results for ALB and CRE concentrations [52]. All components hat were mounted in and on the detection box were controlled by Raspberry Pi.

Immunoassays are a robust method for bacterial detection, offering advantages such as easy readout, simple instrumentation, and non-contact detection; microfluidic chips have played a significant role in combining immunoassays with various analytical platforms, capitalising on their miniaturisation, integration, and automation capabilities over the past few decades [53].

Researchers have introduced a colourimetric immunoassay for diagnostic purposes using Raspberry Pi to manage solutions, rotate the turntable and analyse images. The solutions were controlled automatically via a custom-developed application on Raspberry Pi (4 B) by employing precise peristaltic pumps for tasks like injection, mixing, incubation, washing, and reaction. The Raspberry Pi camera was utilised in a darkroom to capture images of the catalysate, which were subsequently analysed using a self-developed application. All steps involving separation, labelling, catalysis, and detection were executed automatically once the “Start” button on the application was pressed. The experimental results showed that this biosensor successfully detected Salmonella Typhimurium in a range from 1.5 × 10^1^ to 1.5 × 10^7^ CFU/mL within just one hour, achieving a notable lower detection limit of 14 CFU/mL [54].

In the following year, the same research team improved the colourimetric immunoassay to analyse the colour transformation and accurately quantify the presence of target bacteria (Salmonella typhimurium). Hydrogen peroxide (H_2_O_2_) was employed for the purpose of etching silver nanoparticles (AgNPs) in order to release Ag+ ions within the context of the colourimetric immunoassay. The colourimetric signal experienced a significant decrease as a result of the specific and efficient suppression of the peroxidase-like activity of platinum nanoparticles (PtNPs) by Ag+ ions. An alteration in colour was detected on a microfluidic platform that was designed to automate the entire process of bacteria detection. The microfluidic platform comprises several key components, including the microfluidic chip, the stepping motor, the Raspberry Pi, the injection pump, and the camera. These elements together form the internal structure of the platform. The complete bacterial detection procedure was seamlessly carried out within a microfluidic chip placed on a specialised platform with full automation. The Raspberry Pi served as the central control unit for all the electronic components, while an app was developed in the Python environment using the PyCharm IDE and the OpenCV function library. This App encompassed three main functions: firstly, the automatic control of the stepping motor to rotate the columnar chamber and connect the desired channel; secondly, the automated control of the injection pump for tasks such as injection, pipetting and mixing; and thirdly, the automatic collection of tetramethylbenzidine (TMB)-oxide images within the colouring chamber under white LED lighting, followed by the analysis of saturation levels to determine the bacterial concentration [53]. The microfluidic biosensor and the combination of a colourimetric immunoassay with a microfluidic platform proved successful in detecting Salmonella typhimurium at low concentrations [54]. These findings demonstrate the potential of using Raspberry Pi and microfluidics together for detecting various pathogenic bacteria.

Protein C is a proposed biomarker for sepsis, caused by bacteria in the blood. The rapid and accurate detection of Protein C in blood plasma may give information beneficial to patient outcomes. One such microfluidic chip uses immunoassay detection of Protein C. On-chip immobilised metal affinity chromatography (IMAC) was used to separate Protein C from patient plasma. In this system, the camera images fluorescent changes on the chip in ~2 mm diameter circular compartments. The setup utilised a Raspberry Pi and camera connected to a Nikon fluorescence microscope [38]. However, based on the size of the area imaged, the readout could be adapted to a Raspberry Pi camera module, with the addition of excitation LEDs and emission filters.

The Raspberry Pi V2 camera module has been used in many systems [38,47,55,56]. A channel-based system used the Raspberry Pi V2 module to image colourimetric changes in 0.3 mm wide capillaries [7,55]. The camera imaged multiple capillaries at once, capturing the change in resazurin from blue to pink following the growth of bacteria. However, this system did not use any additional lens. Indicating reasonable resolution of the camera module alone on small areas of detection, however, the number of pixels per detection area will be reduced and may introduce more variation in the data. The resolution required for each system needs to be assessed based on each setup.

A sensor system is presented to directly monitor the metabolism of mammalian cells by measuring the uptake of oxygen; it is designed to meet the specific requirements for usage in biolabs. A Raspberry Pi camera is equipped with an optical filter with a cut-off wavelength at 600 nm, and this optical setup combines the camera with an excitation LED. The system fulfils the requirements for reliable oxygen sensing, a small footprint, compatibility with materials, high integration, and automation, achieved through a microfluidic chip with an integrated oxygen-sensitive film, a heater, a temperature sensor, an external optical read-out, and 3D-printed holders and housing. The oxygen-sensitive film decreases phosphorescence with increasing oxygen levels, which were measured using an excitation LED and a Raspberry Pi camera. The chip, fabricated using clean-room technologies, combines silicon and glass, while an excitation LED and a Raspberry Pi camera enable accurate determination of dissolved oxygen concentration within a wide range of oxygen levels and temperatures. The complete chip measures 9.5 mm by 11.5 mm by 0.9 mm^3^ in size. Light intensity measurements for the phosphorescent signal are performed using a small Raspberry Pi camera as the readout mechanism. The closed housing and closed-loop control of the excitation LED are employed to minimise measurement errors, including background light [37]. The optical read-out system, comprising a camera and an excitation LED, offers a cost-effective and accurate solution.

A Raspberry Pi-based device has been developed to predict the relative viscosity of test fluids by measuring the real-time velocity using video analysis [40]. The change in greyscale values within a defined region of interest (ROI) was quantified, enabling the calculation of time and the subsequent determination of viscosity values. The Raspberry Pi was programmed to perform real-time image processing for automated viscosity measurement on paper microstrips with colour pads. It was housed in a 3D-printed box along with a Pi camera module and display, enabling it to be conveniently used as a handheld device. The Raspberry Pi incorporates a microprocessor with a dedicated camera port for real-time video capture and processing. The image analysis was performed on the Raspberry Pi, and the program running on the microprocessor detected and recognized the circular pads, marked in blue and green, located next to the microchannel, which serve as the ROI to be extracted from the video frames. The time taken by the fluid to cover a fixed length between two spots in the microchannel was calculated as he viscosity based on the programmed and color-coded ROIs. The accuracy level of the new Pi-based device was calculated by comparing the viscosity values produced by the device with a benchtop rotational viscometer, and this value was determined to be ±8%, indicating an overall accuracy of 92% [40].

### 3.2. Direct Cellular Image Detection Systems and Microscopy

There is a growing demand for a compact, portable, cost-effective, and high-performance microscope capable of the real-time imaging of cells and lab-on-a-chip devices for use in Point-of-Care diagnostics [60]. However, digital microscopy requires precision hardware, complex components and lighting.

Image sensor-based mini-microscope systems have been developed and successfully utilized within conventional cell culture incubators [30,46]. In one study, a cost-effective and versatile platform was developed for monitoring cell behaviour in an incubator and on lab-on-a-chip devices. With this system, long-term cell behaviour was captured, enabling real-time image processing for the analysis of cell proliferation rates, while the high-speed image-capturing capability of the mini-microscope was demonstrated through the examination of droplet generation in a continuous microfluidic system. A mini-microscope (16 cm × 6 cm × 6 cm), which is equipped with the capability to provide livestream video for remote monitoring, was built using Raspberry Pi Model B+ and a 5-megapixel complementary metal oxide semiconductor (CMOS) a Raspberry Pi camera module. To optimize the imaging capabilities, modifications that enabled the system to achieve a clear field of view and suitable magnification for objects on the scale of tens of micrometres were made to a camera module, including the adoption of an inverse dual-lens system. The recording of video clips is enabled by a Raspberry Pi camera module, offering various frame rates of 7, 30, 60, and 90 frames per second (FPS), and corresponding resolutions of 2592 × 1944, 1920 × 1080, 1280 × 720, and 640 × 480 pixels, respectively [46].

An agglutination lab chip with a lens-free CMOS image sensor, employing the OV8833 sensor from Omni Vision, for example, was developed for POC testing. Deionized water, type A Rhesus (Rh)-positive blood, and type B Rh-positive blood were tested using the CMOS image mini-system, and the Raspberry Pi (3 Model B) was employed to capture and analyse the images of the microfluidic system. The function of this finger-operated chip was demonstrated by blood typing (ABO and Rh). Compared to existing automated blood-analysis methods, the low-cost and portable lab chip combined with CMOS image sensing can easily be performed with very small amounts of blood samples without the need for experienced/trained personnel. While building the agglutination testing system, the researchers aimed to obtain results quickly, minimize the errors of untrained personnel and perform the aforementioned portable agglutination test inexpensively [43].

A flow-rate-based paper microfluidic assay was presented to assess the quantification of whole blood coagulation in a rapid, straightforward, low-volume and cost-effective manner. For monitoring and analysing the assay, researchers developed a device based on Raspberry Pi technology. The device consists of a Raspberry Pi 3, a 7-inch touchscreen display, and a Raspberry Pi camera module, enclosed within a 3D-printed case. The microfluidic device can provide personalized dosing information for anticoagulants and their reversal agents. The device captured images of the paper microfluidic chip at predefined time intervals. These images included overlays with details such as the tested drug, the concentration and the specific time point [44].

Thus, by building lab devices such as image-detection systems and microscopes by making use of open-source hardware, each researcher will be able to build their own equipment, saving time and money.

In some cases, the FOSH version of existing laboratory hardware is developed and used by multiple users for different applications [41]. The OpenFlexure microscope (OFM) has been used to image both bacterial [42] and mammalian cells [61]. The 3D-printed, laboratory-level automated OFM has been designed to increase accessibility to microscopy. The fully 3D-printed design enabled low-volume production and easy maintenance and repairs. OFM has a Raspberry Pi camera V2 (8 MP CMOS sensor) for digital imaging. OFM focuses on delivering accurate 3D movement for the precise focusing and positioning of samples. This is achieved through a thoroughly characterized flexure mechanism, enabling three-axis positioning with exceptionally small step sizes of 50 nm in the *z*-axis and 70 nm in both the x- and y-axes. Although the design has a limited load capacity that is not suitable for very large or heavy samples because of plastic construction, the range of motion and load capacity is sufficient for most microscopy applications. The researchers presented various imaging techniques using the OFM, including brightfield imaging with both epi- and trans-illumination, polarised light microscopy, and multi-channel fluorescence imaging. The OFM obtained high-resolution images of a Giemsa-stained blood smear, enabling the clear identification of individual red blood cells and ring-form trophozoites of Plasmodium falciparum, demonstrating its suitability for malaria diagnosis [41].

OFM project can be considered as a good example in terms of documentation. By creating both a website and a repository on GitLab, the microscope builders have provided easy access to the information needed by anyone who wants to build the OFM. The wealth of content on the site and in the repository includes items such as a bill of materials, assembly instructions with diagrams, STL files, an interactive view of the microscope and software. In this way, anyone interested in the project has access to detailed documentation that guides them step-by-step and allows them to understand the structure and operation of the project. Researchers [42] external to the OFM project have demonstrated the use of OFM for other applications, including evaluating novel antibiotic susceptibility tests. The system used microcapillaries filled with soft agar in the presence and absence of different antibiotics. The OpenFlexure Microscope recorded motile bacteria swimming into the capillaries in the absence of antibiotics, and if susceptible, the bacteria showed a reduced ability to swim into the antibiotic-containing capillaries [42].

Other studies use Raspberry Pi controls to enhance the use of commercial microscopy systems. Cellular imaging and the detection of circulating tumour cells in human whole blood, for example, can be helpful for the early detection of cancer and monitoring the effectiveness of treatments. In the realm of existing imaging flow cytometry, the detection apparatus exhibits substantial size and cost. In order to obtain results, samples must be transported to the laboratory after collection and processed and analysed by trained personnel, followed by long analysis cycles and complex procedures. To address these problems, researchers have proposed a portable, cost-effective, and automated imaging detection platform that combines a microfluidic chip and an image-acquisition module with a portable lens. Samples are made to flow through a channel, and the camera images cells and particles as they flow through. Rather than scatter data collected using traditional flow cytometry, cells are classified using image analysis. The entire system is controlled and the data processing is performed using an embedded Raspberry Pi platform. It is capable of capturing clear images of human blood cells, tumour cells and microspheres of various sizes [45].

In another microscope study, a control was also provided using Arduino in addition to a Raspberry Pi. In this study, the open-source microscope employs a Raspberry Pi equipped with an eight-megapixel Pi camera module V2. Additionally, an Arduino microcontroller is utilised to control the stepper motors and illumination. All operations can be managed either through a keyboard linked to the Raspberry Pi or through a specialised Arduino control interface connected to the mainboard. The primary structure of the microscope is constructed using LEGO bricks. Afterward, a straightforward fluorescence imaging setup was built specifically for capturing images of microfluidic chips. To visualise the chip, a Hayear C-mount objective lens with magnification ranging from 5× to 120×, paired with a 12.3 MP CMOS imager (the Raspberry Pi HQ camera), was used. This setup allowed the researchers to directly capture images within the field of view. In front of the lens, a FITC emission filter (Nikon 515–555 nm) was attached to a 3D-printed housing to illuminate the chip from above [39].

Building a portable and cost-effective cell-detection platform that can be used outside of the lab environment and can complete tasks such as imaging, automated analysis and analysis results access without the need for trained personnel is an ideal approach to addressing challenges related to dependence on trained personnel, high costs, time-consuming and complex test procedures, as well as data analysis and accessibility [45].

High-resolution microscopy to resolve individual cells requires more complex equipment and components, such as lenses, objectives and specific lighting. Even if open-source materials are well documented, such systems are sensitive to small changes in build movements, making it harder for non-experts to build such equipment.

### 3.3. Time-Lapse Imaging

Many experimental systems require or benefit greatly from time-resolved data. Without easily controllable imaging systems, time-series data are highly laborious and time-consuming for the researcher. In many cases, smartphones have been used for capturing and analysis in microfluidic systems [62,63,64,65], but they are impractical for time courses because custom holders need to be used, the systems are not always open-source or they are semi-open systems that combine elements of both open-source and proprietary technologies. Camera settings can be complex to control and are powered with batteries, so they may not be useful for long experimental times. Of note, the Raspberry Pi camera module V2 has similar characteristics to many smartphone cameras; therefore, many experimental setups imaging with a smartphone are likely to be compatible with a Raspberry Pi system. These time-lapse imaging systems need to control the frequency of imaging, different lighting capacities and the control of external light sources, which can be accomplished through simple python code.

While many examples use time series data and capture images using Raspberry Pi and a Pi camera module over time, these are often application-driven. One example is the “PiRamid”, an imaging system that was designed to be used with multiple applications in mind. In a microfluidic example, the PiRamid follows the testing of bacterial growth and antibiotic susceptibility, imaging the colour change of a metabolic dye in microcapillary chips. The PiRamid exploits the use of open-source 3D printing to producean imaging device that is fully customisable and capable of imaging conventional plastic consumables such as microtitre and agar plates alongside commercially available microfluidics such as microcapillary film [47]. The design centres around the simple-to-use and low-cost Raspberry Pi 3 Model B+ single-board computer system and Pi Camera Module v2. In the PiRamid design, the V2 camera is positioned 95 mm away from the subject, resulting in a viewing area for samples measuring 116 mm × 86 mm. These samples are illuminated in brightfield using a white backlight area of 100 × 850 mm. Through the use simple python script programming controlling the camera capture and LED backlight, we see the combination of FOSH and microfluidics producing a compact, low-cost, and high-performance system for automated laboratory imaging. The Raspberry Pi is also capable of directly powering 5 V LEDs; however, for higher voltage lighting, the Pi can be used with separate power supplies and relays. The system is packaged together in a closed imaging box, which stops external light interfering in imaging, making images more reproducible.

### 3.4. Digital Biomolecule Detection: Droplet Microfluidics

Droplet technology is used in many fields, from drug screening to single-cell analysis, DNA/RNA sequencing and the creation of artificial synthetic cells [51]. Imaging of droplet and microcompartments may include colour and fluorescence changes; however, these compartments tend to be very small and differ from other imaging systems, as they often involve flow. Imaging feedback can also be used to control droplet formation.

The behaviour of dispersed droplets in a liquid phase is significant in various industrial and technological applications, including liquid–liquid extraction, emulsion formation, wastewater treatment and hydrometallurgy [48]. Understanding the hydrodynamics of individual droplets and their interactions is essential for improving the efficiency of these processes [48]. In laboratory experiments, controlling the formation of droplets with specific diameters and detachment frequencies is crucial for studying droplet dynamics, collisions and emulsion film formation under dynamic conditions [48]. In recent years, droplet microfluidics has attracted attention in different fields because they offer high-throughput systems and sensors with high framerates per second with low sample usage and reagent-consumption rates. In a conventional droplet microfluidic measurement system, droplets can be observed with a microscope–camera combination [1]. Droplet microfluidic systems tend to become more complex and need customisation; thus, customizable, low-cost and open-source hardware systems can solve this issue [1]. Microfluidic devices have emerged as the preferred method for generating single droplets in a liquid phase, thanks to extensive research and documentation on their fabrication and modification and precise control over droplet size and generation rate [48].

A low-cost, portable device controlled by Raspberry Pi was built to image microfluidic droplets and analyse droplet properties in lab environments [51]. Droplets were generated on a T-shaped chip and flowed into a compartment for imaging and analysis. In order to analyse droplet properties, droplet intensities were plotted against 1/40, 1/20 and 1/10 dilutions of black dye concentrations. A total of about 500–700 droplets were detected in each chamber based on the flow rates they produced. It was observed that at different flow rates of the dye, the droplet size exhibited an increase in diameter, ranging from an average of 78.3 μm to 94.1 μm to 107.6 μm. This low-budget device allows high-throughput droplet production to be observed in microchannels and automated droplet analysis. The setup utilised a Raspberry Pi (3, Model B+) as the main device for executing programs and storing data, a Raspberry Pi camera (Module V2) in conjunction with a macro lens (20×, AUKEY) for capturing detailed images of the microchannels, and an LCD (UCTRONICS 3.5 in. touch screen) for the real-time display of live and recorded images (default resolution of 3280 × 2464) and videos (120 fps for 10 s), with the total cost amounting to approximately 100 USD (in 2019) [51]. In another study, researchers conducted a comparison of three affordable imaging sensors (Raspberry Pi V2, Raspberry Pi HQ and Basler Ace), which are suitable for potential use in droplet microfluidic systems. They evaluated two distinct entry-level open-source systems powered by Raspberry Pi with a higher-priced mid-range Basler camera. The experiments used a mixture of deionised water and fluorescein isothiocyanate-dextran (FITC), a fluorescent dye, to label the droplets. Capturing droplets was successful with all three cameras in terms of sharpness, brightness and droplet condition, indicating that the Raspberry Pi camera modules can be used as a low-cost alternative to the Basler Ace camera module, which is a commercial product manufactured by Basler (>USD 400). The entry-level systems were able to reach 200 and 665 frames per second and the mid-range comparison reference to 750 fps. It should also be noted that all three of the cameras under comparison are significantly cheaper than currently used high-speed cameras [1].

Researchers introduced PortaDrop, which is a portable digital microfluidics platform that uses electrowetting-on-dielectric (EWOD) to manipulate discrete volumes of liquid in the form of droplets. Droplet formation is controlled by the Raspberry Pi 3B+, which communicates with two ATtiny45 microcontrollers via Inter-Integrated Circuit (I2C) to generate the AC high voltage required for droplet activation. The control of the platform is made convenient through a user-friendly 7-inch touchscreen LCD display. PortaDrop incorporates two circuit boards: the mainboard, responsible for controlling all system functionalities and communicating with the Raspberry Pi I2C-Bus, and the secondary board, housing semiconductor switches that enable the activation and deactivation of the electrowetting path electrodes on the bottom chip. In the application, the authors demonstrate a protocol that involves the sequential exchange of droplets using passive dispensing. In this study, a holder is positioned above the EWOD chips to accommodate the eight-megapixel Raspberry Pi camera, which is connected to the camera port of the Raspberry Pi through the Camera Serial Interface (CSI) to record the experimental observations [49].

A microfluidic system for monitoring oil particles was built by incorporating a microfluidic chip, a servo system integrated with microfluidic drive technology, an eight-megapixel Raspberry Pi camera module, and an external macro lens. Specialised image-acquisition software was utilised to capture images of moving oil particles within the microfluidic chip. The hardware component of the system managed the collection and observation of particle images, while the software system was responsible for analysing, processing and identifying these images. The hardware setup consisted of a Raspberry Pi 3B+ integrated into a main board with dimensions of 80 mm × 55 mm × 20 mm. The camera had the capability of capturing still pictures with a resolution of 3280 × 3464 pixels and recording videos in 1080p at a frame rate of 30 fps, as well as 720p videos at 60 fps [50].

These technologies offer high efficiency, resolution and low-cost solutions for various applications, ranging from industrial processes to microbiology and molecular analysis. The use of portable devices controlled by Raspberry Pi enables high-throughput droplet production and automated analysis. These advancements contribute to the improvement of efficiency, accessibility and accuracy in biomolecule detection and analysis.

Few comparisons of camera performance are available for microfluidic systems, due to a lack of standardisation and the non-commercial nature of most research systems. As we have seen, different imaging setups have been used for different devices; however, it can be difficult for researchers building these hardware systems to decide whether these differences are a requirement of the microfluidic system or because of convenience. One study comparing colourimetric absorbance in 0.2 mm capillaries using a range of digital cameras ranging from a mid-range GBP 400 smartphone with a camera to >GBP 5000 laboratory gel:doc system found little difference in absorbance quantitation [66]. However, some of the images from lower-end camera phones (<GBP 50) were able to calculate absorbances but were not able to resolve individual capillaries without additional lenses. Therefore, a less complex system may be used in some cases. However, the required resolution must be assessed for each new system.

## 4. Conclusions

The integration of Raspberry Pi and open hardware with microfluidics has opened up new possibilities for the development of low-cost and customizable systems. The utilisation of Raspberry Pi in direct and indirect image detection and microscopic digital biomolecule detection has significantly contributed to the field of microfluidics. Multiple open-source systems such as Opentrons OT-2 pipetting robot (Opentrons, New York, NY, USA), the OpenFlexure Microscope [67], ImJoy, [68] and UC2 (You. See. Too.) [69] were combined together [70] to develop a complete workflow. FOSS and software for image analysis can be a significant roadblock in the development of fully combined systems. In many of the examples discussed here, images were transferred to another computer and analysed using different software. The choices on the hardware to use for a new build or recreating a system that another researcher has developed and published can be difficult, and standard reporting documentation should be developed and used. For example, studies reporting diagnostic accuracy follow standardised reporting procedures [71].

These advancements offer cost-effective solutions, automation and improved control in imaging. The combination of open-source hardware with microfluidics paves the way for further innovation and the democratisation of this technology, making it more accessible to researchers and practitioners in diverse scientific disciplines.

## Figures and Tables

**Table 1:** Raspberry Pi boards technical specifications.

	Raspberry Pi Zero 2 W	Raspberry Pi 3 Model A+	Raspberry Pi 3 Model B+	Raspberry Pi 4 Model B
Net price *	GBP 17	GBP 25	GBP 40	GBP 35 to GBP 75 (dependent on RAM)
Core type	Cortex-A53 64-bit	ARM Cortex-A53 64-bit	ARM Cortex-A53 64-bit	(ARM v8) Cortex-A72 64-bit
Number of cores	4	4	4	4
GPU speed	1 GHz	1.4 GHz	1.4 GHz	1.5 GHz
RAM	512 MB	512 MB DDR2	1 GB DDR2	1 GB, 2 GB, 4 GB, 8 GB LPDDR4 (depending on model)
USB ports	1× Micro OTG	1× USB 2.0	4× USB 2.0	2× USB3.0 + 2× USB2.0 + USB-C OTG
Ethernet	No	No	10/100/1000 Mbit/s (~300 Mbit/s) Ethernet Port	Gigabit
Wi-Fi	On Board WiFi 802.11n	On Board WiFi 802.11ac Dual Band 2.4 GHz and 5 GHz	On Board WiFi 802.11ac Dual Band 2.4 GHz and 5 GH	On Board WiFi 802.11ac Dual Band 2.4 GHz/5 GHz
Bluetooth	4.2 BLE	4.2 BLE	2.0/4.1/4.2 LS BLE	5.0 BLE
HDMI port	Mini HDMI	Full HDMI	Full HDMI	2× micro HDMI (up to 4kp60 supported)
Video output	Mini HDMI Composite via PCB	HDMI 3.5 mm Composite DSI (for LCD)	HDMI 3.5 mm Composite DSI (for LCD)	HDMI 3.5 mm Composite DSI (for LCD)
Camera input	No	15 Pin CSI	15 Pin CSI	15 Pin CSI
Number of GPIO pins	40	40	40	40
GPIO functions	17 × GPIO UART I^2^C SPI I^2^S 1-Wire 3.3 V/5 V/GND EEPROM	17 × GPIO UART I^2^C SPI I^2^S 1-Wire 3.3 V/5 V/GND EEPROM	17 × GPIO UART I^2^C SPI I^2^S 1-Wire 3.3 V/5 V/GND EEPROM	17 × GPIO UART I^2^C SPI I^2^S 1-Wire 3.3 V/5 V/GND EEPROM
Memory	MicroSD			
Power input	Micro USB, GPIO 5 V @ 2.5 A	Micro USB, GPIO 5 V @ 2.5 A	Micro USB, GPIO 5 V @ 2.5 A	USB-C, GPIO 5 V @ 1.25 A

* Price as of 4 October 23.

**Table 2:** Raspberry Pi Camera modules specifications.

	Camera Module v2	Camera Module 3	High Quality (HQ) Camera
Net price *	GBP 28.50	GBP 26	GBP 57
Size	≅25 × 24 × 9 mm	≅25 × 24 × 11.5 mm	≅38 × 38 × 18.4 mm
Lens mount	N/A	N/A	C/CS- or M12-mount
Still resolution	8 Megapixels	11.9 Megapixels	12.3 Megapixels
Video modes	1080p47, 1640 × 1232p41 and 640 × 480p206	2304 × 1296p56, 2304 × 1296p30 HDR, 1536 × 864p120	2028 × 1080p50, 2028 × 1520p40 and 1332 × 990p120
Sensor	Sony IMX219	Sony IMX708	Sony IMX477
Sensor resolution	3280 × 2464 pixels	4608 × 2592 pixels	4056 × 3040 pixels
Sensor image area	3.68 × 2.76 mm (4.6 mm diagonal)	6.45 × 3.63 mm (7.4 mm diagonal)	6.287 mm × 4.712 mm (7.9 mm diagonal)
Pixel size	1.12 µm × 1.12 µm	1.4 µm × 1.4 µm	1.55 µm × 1.55 µm
Optical size	1/4”	1/2.43”	1/2.3”
Horizontal Field of View (FoV)	62.2 degrees	66 degrees	Depends on lens
Vertical Field of View (FoV)	48.8 degrees	41 degrees	Depends on lens
Focus	Adjustable	Motorised	Adjustable
Focal ratio (F-Stop)	F2.0	F1.8	Depends on lens
Maximum Exposure times (seconds)	11.76	112	670.74

* Price as of 4 October 23 [35,36].

**Table 3:** Examples that illustrate the diverse uses of Raspberry Pi in open-source imaging hardware.

Category	Example Application	Raspberry Pi Model (Where Stated)	Camera Model	Example Camera Image Specifications	Example Reference
Colour and Fluorescence detection	paper microfluidic system to detect albumin-to-creatine ratio in urine	4 B	V2		[52]
	colourimetric immunoassay	4 B	Pi camera		[53,54]
	colourimetric immunoassay		V2		[7,38,47,55,56]
	real-time viscosity	zero			[40]
Direct Cellular Image Detection Systems and Microscopy	mini microscope	B+	5-MP Pi camera	video clips fps of 7, 30, 60, and 90, and corresponding resolutions of 2592 × 1944, 1920 × 1080, 1280 × 720, and 640 × 480 pixels, respectively	[46]
	agglutination testing system	3 B	-		[43]
	OpenFlexure Microscope		V2		[41,42]
	microscope		V2 and HQ		[39]
	Planktoscope: direct plankton imaging	4 B	V2 and HQ with M12 Lens 16 MM 5 MP 1/2.5”		[57]
Time-Lapse Imaging	bacterial growth and antibiotic susceptibility testing	3 B+	V2		[47]
Digital Biomolecule Detection: Droplet Microfluidics	microfluidic droplets	3 B+	V2	default resolution: 3280 × 2464 and videos: 120 fps for 10 s	[11]
	portable digital microfluidics platform	3 B+	V2		[49]
	monitoring oil particles	3 B+	V2	still pictures resolution: 3280 × 3464 pixels and 1080p videos at 30 fps, 720p videos at 60 fps	[50]

## References

[1] Parnamets K., Koel A., Pardy T., Rang T. Open Source Hardware Cost-Effective Imaging Sensors for High-Throughput Droplet Microfluidic Systems. Proceedings of the 2022 26th International Conference Electronics.

[2] Nuñez I., Matute T., Herrera R., Keymer J., Marzullo T., Rudge T., Federici F. (2017). Low cost and open source multi-fluorescence imaging system for teaching and research in biology and bioengineering. PLoS ONE.

[3] Pearce J.M. (2014). Laboratory equipment: Cut costs with open-source hardware. Nature.

[4] Baden T., Chagas A.M., Gage G., Marzullo T., Prieto-Godino L.L., Euler T. (2015). Open Labware: 3-D Printing Your Own Lab Equipment. PLoS Biol..

[5] Pearce J.M. (2017). Impacts of Open Source Hardware in Science and Engineering. Bridge.

[6] Pearce J.M. (2020). Economic savings for scientific free and open source technology: A review. HardwareX.

[7] Needs S.H., Diep T.T., Bull S.P., Lindley-Decaire A., Ray P., Edwards A.D. (2019). Exploiting open source 3D printer architecture for laboratory robotics to automate high-throughput time-lapse imaging for analytical microbiology. PLoS ONE.

[8] Griffin K., Pappas D. (2023). 3D printed microfluidics for bioanalysis: A review of recent advancements and applications. TrAC Trends Anal. Chem..

[9] He Y., Wu Y., Fu J., Gao Q., Qiu J. (2016). Developments of 3D Printing Microfluidics and Applications in Chemistry and Biology: A Review. Electroanalysis.

[10] Nielsen A.V., Beauchamp M.J., Nordin G.P., Woolley A.T. (2020). 3D Printed Microfluidics. Annu. Rev. Anal. Chem..

[11] Su R., Wang F., McAlpine M.C. (2023). 3D printed microfluidics: Advances in strategies, integration, and applications. Lab Chip.

[12] Maia Chagas A., Molloy J.C., Prieto-Godino L.L., Baden T. (2020). Leveraging open hardware to alleviate the burden of COVID-19 on global health systems. PLoS Biol..

[13] Cardoso R.M., Santos R.O., Munoz R.A.A., Garcia C.D., Blanes L. (2020). A Multi-Pump Magnetohydrodynamics Lab-On-A-Chip Device for Automated Flow Control and Analyte Delivery. Sensors.

[14] da Costa E.T., Mora M.F., Willis P.A., do Lago C.L., Jiao H., Garcia C.D. (2014). Getting started with open-hardware: Development and control of microfluidic devices. Electrophoresis.

[15] Kehl F., Cretu V.F., Willis P.A. (2021). Open-source lab hardware: A versatile microfluidic control and sensor platform. HardwareX.

[16] Lane S.I.R., Butement J., Harrington J., Underwood T., Shrimpton J., West J. (2019). Perpetual sedimentation for the continuous delivery of particulate suspensions. Lab Chip.

[17] Soenksen L.R., Kassis T., Noh M., Griffith L.G., Trumper D.L. (2018). Closed-loop feedback control for microfluidic systems through automated capacitive fluid height sensing. Lab Chip.

[18] Watson C., Senyo S. (2019). All-in-one automated microfluidics control system. HardwareX.

[19] White J.A., Streets A.M. (2018). Controller for microfluidic large-scale integration. HardwareX.

[20] Zhu H., Özkayar G., Lötters J., Tichem M., Ghatkesar M.K. (2023). Portable and integrated microfluidic flow control system using off-the-shelf components towards organs-on-chip applications. Biomed. Microdevices.

[21] Vo P.Q.N., Husser M.C., Ahmadi F., Sinha H., Shih S.C.C. (2017). Image-based feedback and analysis system for digital microfluidics. Lab Chip.

[22] Prabhu G.R.D., Urban P.L. (2020). Elevating Chemistry Research with a Modern Electronics Toolkit. Chem. Rev..

[23] Kassis T., Perez P.M., Yang C.J.W., Soenksen L.R., Trumper D.L., Griffith L.G. (2018). PiFlow: A biocompatible low-cost programmable dynamic flow pumping system utilizing a Raspberry Pi Zero and commercial piezoelectric pumps. Hardwarex.

[24] Lake J.R., Heyde K.C., Ruder W.C. (2017). Low-cost feedback-controlled syringe pressure pumps for microfluidics applications. PLoS ONE.

[25] Loy D.M., Krzyszton R., Lachelt U., Radler J.O., Wagner E. (2021). Controlling Nanoparticle Formulation: A Low-Budget Prototype for the Automation of a Microfluidic Platform. Processes.

[26] Smyth J., Smith K., Nagrath S., Oldham K. Modeling, Identification, and Flow Control for a Microfluidic Device using a Peristaltic Pump. Proceedings of the 2020 American Control Conference (ACC).

[27] Wijnen B., Hunt E.J., Anzalone G.C., Pearce J.M. (2014). Open-Source Syringe Pump Library. PLoS ONE.

[28] Winkler S., Menke J., Meyer K.V., Kortmann C., Bahnemann J. (2022). Automation of cell culture assays using a 3D-printed servomotor-controlled microfluidic valve system. Lab Chip.

[29] Zhang Y., Tseng T.-M., Schlichtmann U. (2021). Portable all-in-one automated microfluidic system (PAMICON) with 3D-printed chip using novel fluid control mechanism. Sci. Rep..

[30] Maia Chagas A., Prieto-Godino L.L., Arrenberg A.B., Baden T. (2017). The €100 lab: A 3D-printable open-source platform for fluorescence microscopy, optogenetics, and accurate temperature control during behaviour of zebrafish, Drosophila, and Caenorhabditis elegans. PLoS Biol..

[31] Damborsky P., Svitel J., Katrlik J. (2016). Optical biosensors. Essays Biochem..

[32] Jolles J.W. (2021). Broad-scale applications of the Raspberry Pi: A review and guide for biologists. Methods Ecol. Evol..

[33] Raspberry Pi Trading Ltd. Products. https://www.raspberrypi.com/products/.

[34] Raspberry Pi Trading Ltd. Raspberry Pi Documentation. https://www.raspberrypi.com/documentation/computers/raspberry-pi.html.

[35] The Pit Hut. https://thepihut.com/.

[36] Raspberry Pi Trading Ltd. Raspberry Pi Documentation-Camera. https://www.raspberrypi.com/documentation/accessories/camera.html.

[37] Bunge F., van den Driesche S., Waespy M., Radtke A., Belge G., Kelm S., Waite A.M., Mirastschijski U., Vellekoop M.J. (2019). Microfluidic oxygen sensor system as a tool to monitor the metabolism of mammalian cells. Sens. Actuators B-Chem..

[38] Damodara S., Arora J., Dwivedi D.J., Liaw P.C., Fox-Robichaud A.E., Selvaganapathy P.R., Canadian Critical Care Translational Biology G. (2022). Microfluidic device for single step measurement of protein C in plasma samples for sepsis prognosis. Lab Chip.

[39] Gervais T., Temiz Y., Aubé L., Delamarche E. (2022). Large-Scale Dried Reagent Reconstitution and Diffusion Control Using Microfluidic Self-Coalescence Modules. Small.

[40] Puneeth S.B., Munigela N., Puranam S.A., Goel S. (2020). Automated Mini-Platform With 3-D Printed Paper Microstrips for Image Processing-Based Viscosity Measurement of Biological Samples. IEEE Trans. Electron Devices.

[41] Collins J.T., Knapper J., Stirling J., Mduda J., Mkindi C., Mayagaya V., Mwakajinga G.A., Nyakyi P.T., Sanga V.L., Carbery D. (2020). Robotic microscopy for everyone: The OpenFlexure microscope. Biomed. Opt. Express.

[42] Diep T.T., Needs S.H., Bizley S., Edwards A.D. (2022). Rapid Bacterial Motility Monitoring Using Inexpensive 3D-Printed OpenFlexure Microscopy Allows Microfluidic Antibiotic Susceptibility Testing. Micromachines.

[43] Lu C.H., Shih T.-S., Shih P.-C., Pendharkar G.P., Liu C.-E., Chen C.-K., Hsu L., Chang H.-Y., Yang C.-L., Liu C.-H. (2020). Finger-powered agglutination lab chip with CMOS image sensing for rapid point-of-care diagnosis applications. Lab Chip.

[44] Sweeney R.E., Nguyen V., Alouidor B., Budiman E., Wong R.K., Yoon J.Y. (2019). Flow Rate and Raspberry Pi-Based Paper Microfluidic Blood Coagulation Assay Device. IEEE Sens. J..

[45] Wang R., Huang X., Xu X., Sun J., Zheng S., Ke X., Yao J., Han W., Wei M., Chen J. (2022). A Standalone and Portable Microfluidic Imaging Detection System With Embedded Computing for Point-of-Care Diagnostics. IEEE Sens. J..

[46] Wang Z., Boddeda A., Parker B., Samanipour R., Ghosh S., Menard F., Kim K. (2018). A High-Resolution Minimicroscope System for Wireless Real-Time Monitoring. IEEE Trans. Biomed. Eng..

[47] Long M.M., Diep T.T., Needs S.H., Ross M.J., Edwards A.D. (2022). PiRamid: A compact Raspberry Pi imaging box to automate small-scale time-lapse digital analysis, suitable for laboratory and field use. HardwareX.

[48] Gawel D., Zawala J. (2019). Automatic Single Droplet Generator with Control over Droplet Size and Detachment Frequency. Colloids Interfaces.

[49] Kremers T., Thelen S., Bosbach N., Schnakenberg U. (2020). PortaDrop: A portable digital microfluidic platform providing versatile opportunities for Lab-On-A-Chip applications. PLoS ONE.

[50] Liu Z., Liu Y., Zuo H., Wang H., Fei H., Jiang Z. A Microfluidic Oil Particles Monitoring System based on Raspberry Pi. Proceedings of the 2022 Global Reliability and Prognostics and Health Management (PHM-Yantai).

[51] Sun M., Li Z., Yang Q. (2019). μdroPi: A Hand-Held Microfluidic Droplet Imager and Analyzer Built on Raspberry Pi. J. Chem. Educ..

[52] Chen S.J., Tseng C.C., Huang K.H., Chang Y.C., Fu L.M. (2022). Microfluidic Sliding Paper-Based Device for Point-of-Care Determination of Albumin-to-Creatine Ratio in Human Urine. Biosensors.

[53] Duan H., Qi W., Wang S., Zheng L., Yuan J., Lin J. (2022). Sample-in-answer-out colorimetric detection of Salmonella typhimurium using non-enzymatic cascade amplification. Anal. Chim. Acta.

[54] Qi W., Zheng L., Wang S., Huang F., Liu Y., Jiang H., Lin J. (2021). A microfluidic biosensor for rapid and automatic detection of Salmonella using metal-organic framework and Raspberry Pi. Biosens. Bioelectron..

[55] Needs S.H., Saiprom N., Rafaque Z., Imtiaz W., Chantratita N., Runcharoen C., Thammachote J., Anun S., Peacock S.J., Ray P. (2022). Miniaturised broth microdilution for simplified antibiotic susceptibility testing of Gram negative clinical isolates using microcapillary devices. Analyst.

[56] Schade F., Schwack W., Demirbas Y., Morlock G.E. (2021). Open-source all-in-one LabToGo Office Chromatography. Anal. Chim. Acta.

[57] Pollina T., Larson A.G., Lombard F., Li H., Le Guen D., Colin S., de Vargas C., Prakash M. (2022). PlanktoScope: Affordable Modular Quantitative Imaging Platform for Citizen Oceanography. Front. Mar. Sci..

[58] Gevaerd A., Watanabe E.Y., Belli C., Marcolino-Junior L.H., Bergamini M.F. (2021). A complete lab-made point of care device for non-immunological electrochemical determination of cortisol levels in salivary samples. Sens. Actuators B Chem..

[59] Needs S.H., Pivetal J., Hayward J., Kidd S.P., Lam H., Diep T., Gill K., Woodward M., Reis N.M., Edwards A.D. (2023). Moving microcapillary antibiotic susceptibility testing (mcAST) towards the clinic: Unravelling kinetics of detection of uropathogenic *E. coli*, mass-manufacturing and usability for detection of urinary tract infections in human urine. Sens. Diagn..

[60] Bohidar P., Gupta S., Banerjee I., Pal K., Kraatz H.-B., Khasnobish A., Bag S., Banerjee I., Kuruganti U. (2019). 18—Trends in point-of-care microscopy. Bioelectronics and Medical Devices.

[61] McDermott S., Ayazi F., Collins J., Knapper J., Stirling J., Bowman R., Cicuta P. (2022). Multi-modal microscopy imaging with the OpenFlexure Delta Stage. Opt. Express.

[62] Dönmez S.İ., Needs S.H., Osborn H.M.I., Edwards A.D. (2020). Label-free smartphone quantitation of bacteria by darkfield imaging of light scattering in fluoropolymer micro capillary film allows portable detection of bacteriophage lysis. Sens. Actuators B Chem..

[63] Huang X., Xu D., Chen J., Liu J., Li Y., Song J., Ma X., Guo J. (2018). Smartphone-based analytical biosensors. Analyst.

[64] Needs S.H., Osborn H.M.I., Edwards A.D. (2021). Counting bacteria in microfluidic devices: Smartphone compatible ‘dip-and-test’ viable cell quantitation using resazurin amplified detection in microliter capillary arrays. J. Microbiol. Methods.

[65] Needs S.H., Sirivisoot S., Jegouic S., Prommool T., Luangaram P., Srisawat C., Sriraksa K., Limpitikul W., Mairiang D., Malasit P. (2022). Smartphone multiplex microcapillary diagnostics using Cygnus: Development and evaluation of rapid serotype-specific NS1 detection with dengue patient samples. PLoS Neglected Trop. Dis..

[66] Jegouic S.M., Jones I.M., Edwards A.D. (2021). Affordable mobile microfluidic diagnostics: Minimum requirements for smartphones and digital imaging for colorimetric and fluorometric anti-dengue and anti-SARS-CoV-2 antibody detection. Wellcome Open Res..

[67] OpenFlexure/Openflexure-Microscope-Server GitLab. https://gitlab.com/openflexure/openflexure-microscope-server.

[68] Ouyang W., Mueller F., Hjelmare M., Lundberg E., Zimmer C. (2019). ImJoy: An open-source computational platform for the deep learning era. Nat. Methods.

[69] Diederich B., Lachmann R., Carlstedt S., Marsikova B., Wang H., Uwurukundo X., Mosig A.S., Heintzmann R. (2020). A versatile and customizable low-cost 3D-printed open standard for microscopic imaging. Nat. Commun..

[70] Ouyang W., Bowman R.W., Wang H., Bumke K.E., Collins J.T., Spjuth O., Carreras-Puigvert J., Diederich B. (2022). An Open-Source Modular Framework for Automated Pipetting and Imaging Applications. Adv. Biol..

[71] Cohen J.F., Korevaar D.A., Altman D.G., Bruns D.E., Gatsonis C.A., Hooft L., Irwig L., Levine D., Reitsma J.B., de Vet H.C. (2016). STARD 2015 guidelines for reporting diagnostic accuracy studies: Explanation and elaboration. BMJ Open.

